# Supplementary material for: Parallel within-host evolution alters virulence factors in an opportunistic Klebsiella pneumoniae during a hospital outbreak
Source: Nat Commun. 2025 Sep 30;16:8727. doi: 10.1038/s41467-025-64521-9 (PMC12485182; doi:10.1038/s41467-025-64521-9)
Supplement: Supplementary file 1 — Supplementary Information [file 41467_2025_64521_MOESM1_ESM.pdf]

Supplementary Information

Parallel within-host evolution alters key virulence factors
in an opportunistic *K. pneumoniae* clone during a hospital outbreak

Greta Zaborskytė, Karin Hjort, Birgitta Lytsy, Linus Sandegren

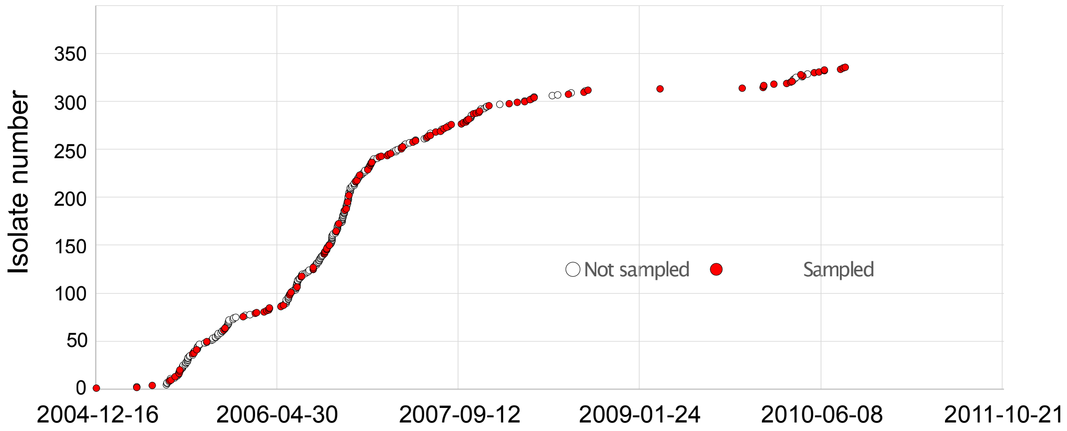

Supplementary Fig. 1 | Cumulative sampling graph of isolates over time of the outbreak. Red circles denote sampled isolates in this study.

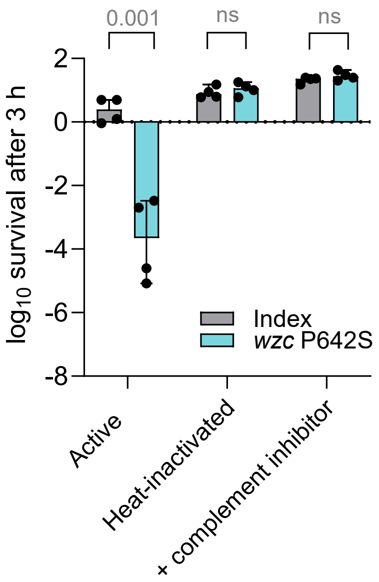

Supplementary Fig. 2 | Serum killing control with active serum, heat-inactivated serum and complement inhibitor polyanetholesulfonic acid. Results from four biological replicates with 95% CI are shown. Difference between the index isolate and *wzc* P642S mutant in each condition tested by unpaired t-test, ns - not significant.

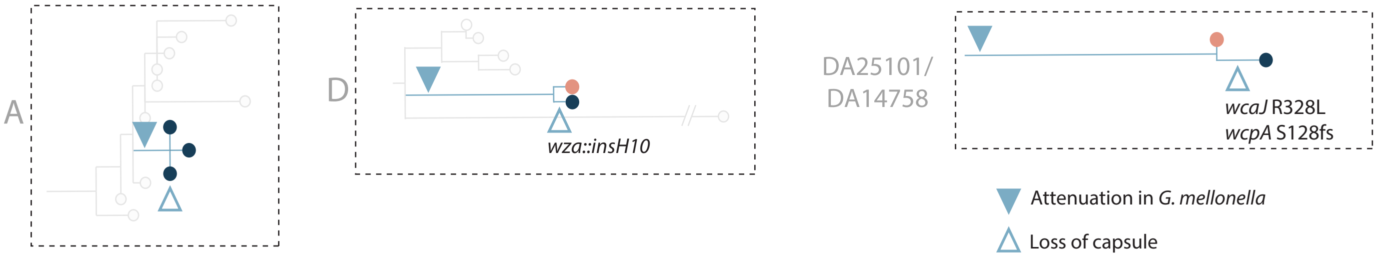

Supplementary Fig. 3 | Phylogenetic indications of mutations reducing killing of *G. mellonella* (blue triangles) and capsule-loss mutations (white triangles) in clusters A, D, and DA25101/DA14758.

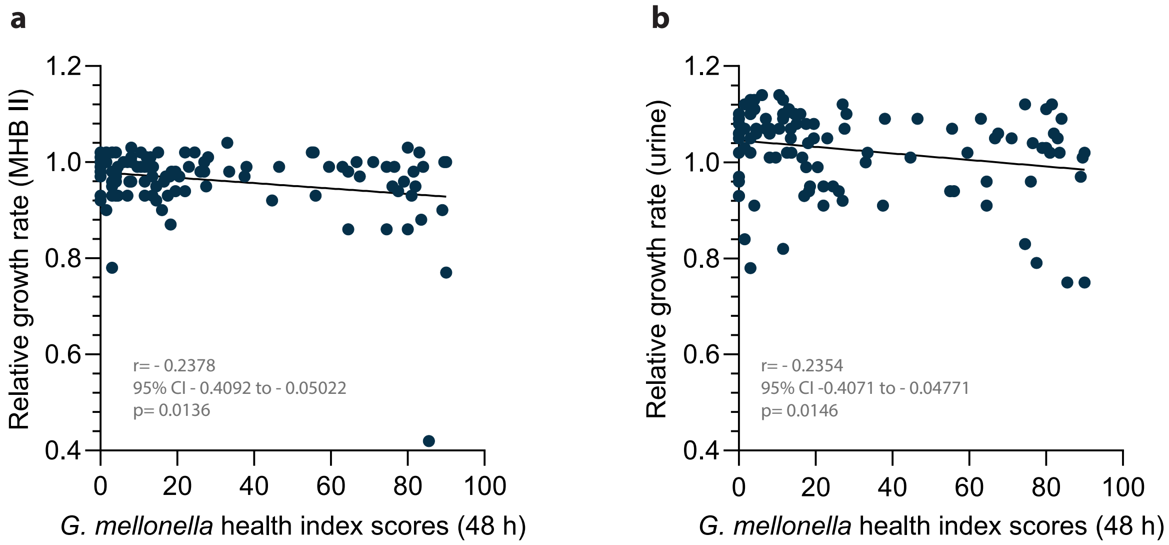

Supplementary Fig. 4 | Exponential growth rates in (**a**) MHB II medium and (**b**) urine vs health index scores in *G. mellonella* larvae at 48 h post-injection. Exponential growth rates refer to means of five biological replicates. Pearson correlation coefficients (r) with 95% CI and a two-tailed p value are shown.

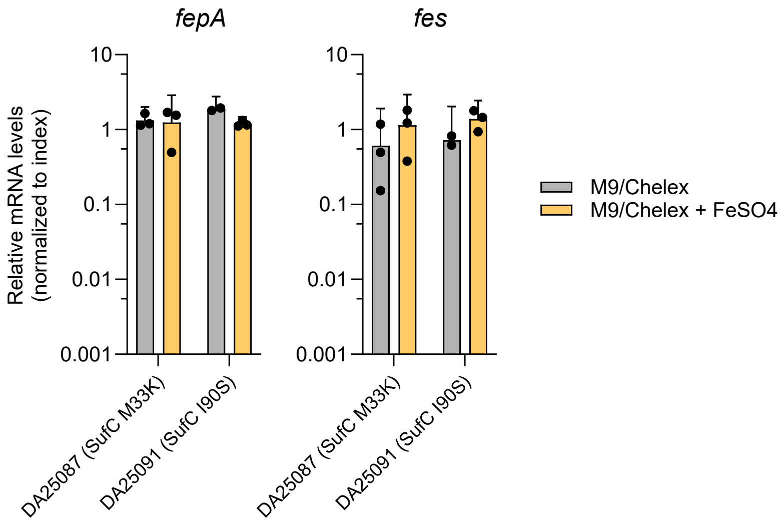

Supplementary Fig. 5 | mRNA levels of *fepA* and *fes* in control isolates with *sufC* mutations but without any *fepA/fes* changes relative to the index isolate. mRNA levels (mean of n=3 with 95% CI) are relative to the index isolate for the respective conditions. Comparison to the index isolate was done by an unpaired t-test with Bonferroni corrections for two comparisons; none of the comparisons were significant.

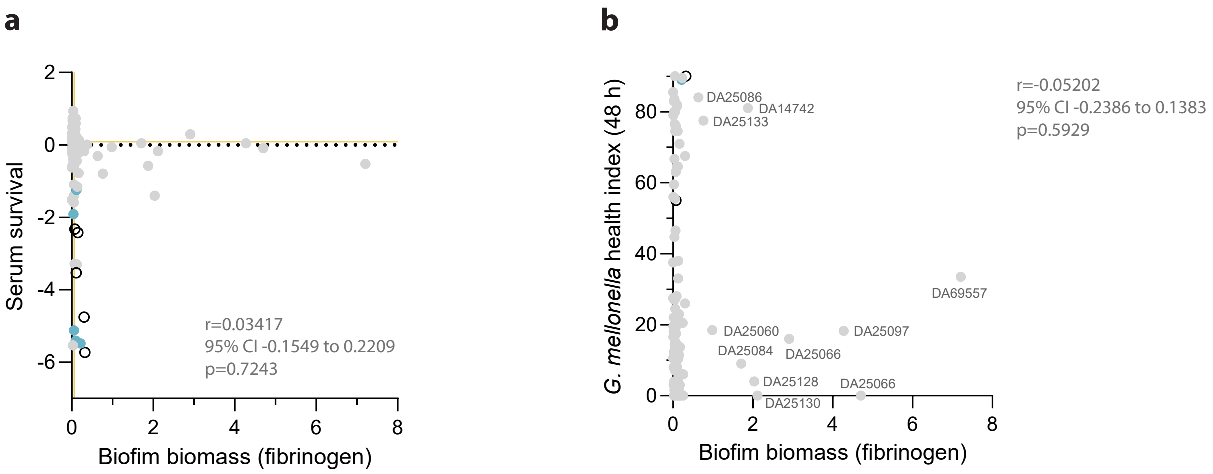

Supplementary Fig. 6 | Interconnection between biofilm biomass on silicone with fibrinogen and (**a**) survival in human serum and (**b**) health index scores of *G. mellonella* at 48 h. Blue dots indicate isolates with *wzc* missense mutations. Pearson correlation coefficients (r) with 95% CI and two-tailed p values is shown.
