# Supplementary material for: Parallel within-host evolution alters virulence factors in an opportunistic Klebsiella pneumoniae during a hospital outbreak
Source: Nat Commun. 2025 Sep 30;16:8727. doi: 10.1038/s41467-025-64521-9 (PMC12485182; doi:10.1038/s41467-025-64521-9)
Supplement: Supplementary file 2 — Description of Additional Supplementary File [file 41467_2025_64521_MOESM2_ESM.pdf]

Description of Additional Supplementary Files

Supplementary Data 1:

List of genetic changes in each isolate

Supplementary Data 2:

List of all genetic changes according to genome position

Supplementary Data 3 :

(dN/dS calculations and list of all genes and intergenic regions with at least 2 independent mutations

Supplementary Data 4 :

Genetic changes associated with virulence by neighbour comparison

Supplementary Data 5 :

Mutations in genes affecting capsule and LPS in isolates.

Supplementary Data 6 :

Connection between iron-related genetic changes and growth in iron-depleted and repleted conditions
