# Supplementary material for: Parallel within-host evolution alters virulence factors in an opportunistic Klebsiella pneumoniae during a hospital outbreak
Source: Nat Commun. 2025 Sep 30;16:8727. doi: 10.1038/s41467-025-64521-9 (PMC12485182; doi:10.1038/s41467-025-64521-9)
Supplement: Supplementary file 9 — Reporting summary [file 41467_2025_64521_MOESM9_ESM.pdf]

nature portfolio

Corresponding author(s): Linus Sandegren

Last updated by author(s): Aug 18, 2025

Reporting Summary

Nature Portfolio wishes to improve the reproducibility of the work that we publish. This form provides structure for consistency and transparency in reporting. For further information on Nature Portfolio policies, see our Editorial Policies and the Editorial Policy Checklist.

Please do not complete any field with "not applicable" or n/a. Refer to the help text for what text to use if an item is not relevant to your study.
For final submission: please carefully check your responses for accuracy; you will not be able to make changes later.

Statistics

For all statistical analyses, confirm that the following items are present in the figure legend, table legend, main text, or Methods section.

n/a	Confirmed	
☐	☒	The exact sample size (*n*) for each experimental group/condition, given as a discrete number and unit of measurement
☐	☒	A statement on whether measurements were taken from distinct samples or whether the same sample was measured repeatedly
☐	☒	The statistical test(s) used AND whether they are one- or two-sided<br>*Only common tests should be described solely by name; describe more complex techniques in the Methods section.*
☐	☒	A description of all covariates tested
☐	☒	A description of any assumptions or corrections, such as tests of normality and adjustment for multiple comparisons
☐	☒	A full description of the statistical parameters including central tendency (e.g. means) or other basic estimates (e.g. regression coefficient) AND variation (e.g. standard deviation) or associated estimates of uncertainty (e.g. confidence intervals)
☐	☒	For null hypothesis testing, the test statistic (e.g. *F*, *t*, *r*) with confidence intervals, effect sizes, degrees of freedom and *P* value noted<br>*Give P values as exact values whenever suitable.*
☒	☐	For Bayesian analysis, information on the choice of priors and Markov chain Monte Carlo settings
☒	☐	For hierarchical and complex designs, identification of the appropriate level for tests and full reporting of outcomes
☐	☒	Estimates of effect sizes (e.g. Cohen's *d*, Pearson's *r*), indicating how they were calculated

Our web collection on statistics for biologists contains articles on many of the points above.

Software and code

Policy information about availability of computer code

Data collection	No specific code was used for data collection.
Data analysis	Genomic analysis was performed using CLC Genomics Workbench v.25 (Qiagen). Statistical analyses were performed using GraphPad Prism version 9.2.0. for macOS (GraphPad Software, San Diego, California, USA).

For manuscripts utilizing custom algorithms or software that are central to the research but not yet described in published literature, software must be made available to editors and reviewers. We strongly encourage code deposition in a community repository (e.g. GitHub). See the Nature Portfolio guidelines for submitting code & software for further information.

Data

Policy information about availability of data

All manuscripts must include a data availability statement. This statement should provide the following information, where applicable:

- Accession codes, unique identifiers, or web links for publicly available datasets
- A description of any restrictions on data availability
- For clinical datasets or third party data, please ensure that the statement adheres to our policy

The sequencing data generated in this study has been deposited at NCBI under bio project number PRJNA857654. Source data are provided with this paper and via figshare (https://doi.org/10.6084/m9.figshare.28678271). Biological material is available from the corresponding author upon reasonable request.

Research involving human participants, their data, or biological material

Policy information about studies with human participants or human data. See also policy information about sex, gender (identity/presentation), and sexual orientation and race, ethnicity and racism.

Reporting on sex and gender	The sex of the patients from which bacteria were isolated was recorded but not used further in any analyses.
Reporting on race, ethnicity, or other socially relevant groupings	These parameters were not recorded.
Population characteristics	No population characteristics of the patients from which bacteria were isolated was used in the study.
Recruitment	Bacterial isolates from patients that suffered from infection or colonization with the particular outbreak clone of K. pneumoniae were included in the study. No consent was needed from the patients since this regards routine isolates without any human material.
Ethics oversight	Permission to include basic patient details linked to bacterial isolates was approved by the Regional Ethics Review Board in Uppsala (Dnr 2017/160).

Note that full information on the approval of the study protocol must also be provided in the manuscript.

Field-specific reporting

Please select the one below that is the best fit for your research. If you are not sure, read the appropriate sections before making your selection.

☒ Life sciences ☐ Behavioural & social sciences ☐ Ecological, evolutionary & environmental sciences

For a reference copy of the document with all sections, see nature.com/documents/nr-reporting-summary-flat.pdf

Life sciences study design

All studies must disclose on these points even when the disclosure is negative.

Sample size	For clinical isolates we used all available isolates in accordance with the number of patients suffering from the clone during the outbreak.
Data exclusions	No data was excluded.
Replication	Al biological and technical replicates were included in the statistical calculations and are indicated in the respective figure legend.
Randomization	All bacterial isolates were tested in the same way. Aloocation to groups was done for statistical testing and then the original isolation sites determined the grouping.
Blinding	Blinding was not used throughout the study. Experiments were performed the same for all isolates and they were not grouped while doing so.

Reporting for specific materials, systems and methods

We require information from authors about some types of materials, experimental systems and methods used in many studies. Here, indicate whether each material, system or method listed is relevant to your study. If you are not sure if a list item applies to your research, read the appropriate section before selecting a response.

Materials & experimental systems

n/a	Involved in the study	
☒	☐	Antibodies
☒	☐	Eukaryotic cell lines
☒	☐	Palaeontology and archaeology
☐	☒	Animals and other organisms
☒	☐	Clinical data
☐	☒	Dual use research of concern
☒	☐	Plants

Methods

n/a	Involved in the study	
☒	☐	ChIP-seq
☒	☐	Flow cytometry
☒	☐	MRI-based neuroimaging

Animals and other research organisms

Policy information about studies involving animals; ARRIVE guidelines recommended for reporting animal research, and Sex and Gender in Research

Laboratory animals	6-8 weeks old BALB/c female mice
Wild animals	The study did not involve wild animals.
Reporting on sex	Use of only female mice is standard for this kind of study.
Field-collected samples	The study did not involve field-collected samples.
Ethics oversight	The study was approved by approved by the Regional Ethics Review Board in Uppsala (Dnr 5.8.18-15552/2019).

Note that full information on the approval of the study protocol must also be provided in the manuscript.

Dual use research of concern

Policy information about dual use research of concern

Hazards

Could the accidental, deliberate or reckless misuse of agents or technologies generated in the work, or the application of information presented in the manuscript, pose a threat to:

No	Yes	
☒	☐	Public health
☒	☐	National security
☒	☐	Crops and/or livestock
☒	☐	Ecosystems
☒	☐	Any other significant area

Experiments of concern

Does the work involve any of these experiments of concern:

No	Yes	
☒	☐	Demonstrate how to render a vaccine ineffective
☐	☒	Confer resistance to therapeutically useful antibiotics or antiviral agents
☐	☒	Enhance the virulence of a pathogen or render a nonpathogen virulent
☒	☐	Increase transmissibility of a pathogen
☒	☐	Alter the host range of a pathogen
☒	☐	Enable evasion of diagnostic/detection modalities
☒	☐	Enable the weaponization of a biological agent or toxin
☒	☐	Any other potentially harmful combination of experiments and agents

Precautions and benefits

Biosecurity precautions	All laboratory precedures were performed i BioSafetyLevel 2 (BSL2) facilities at Uppsala University, Sweden. No redaction of the results have been made.
Biosecurity oversight	The facilities are inspected and routines evaluated yearly by the department safety officers. The BSL2 permit is controlled by the Swedish board of agriculture.
Benefits	The study reveals how opportunistic pathogens like Klebsiella pneumoniae evolves in the human host and describes effects and the underlying genetic changes that happened in a single clone during a hospital outbreak. The study will increase our understanding of how bacterial populations naturally change and how this affects the virulence of the bacteria. No experiments have been performed to increase virulence, only description of what has happened in the patients. Mostly this has resulted in reduced virulence.
Communication benefits	The risk of communicating the results from the studyis deemed very small. We do find chenges that directly affects virulence (reductions) and that identify what genetic factors contributes to this, but this is very unlikely to be contributing to any risk of malifiscent use in the future. By understanding the genetic and evolutionary factors underlying bacterial adaptation in the host, we could instead identify potential variants of concern faster in the future which would help vulnerable patients n thefuture.

Plants

Seed stocks

Novel plant genotypes

Authentication

This checklist template is licensed under a Creative Commons Attribution 4.0 International License, which permits use, sharing, adaptation, distribution and reproduction in any medium or format, as long as you give appropriate credit to the original author(s) and the source, provide a link to the Creative Commons license, and indicate if changes were made. The images or other third party material in this article are included in the article's Creative Commons license, unless indicated otherwise in a credit line to the material. If material is not included in the article's Creative Commons license and your intended use is not permitted by statutory regulation or exceeds the permitted use, you will need to obtain permission directly from the copyright holder. To view a copy of this license, visit http://creativecommons.org/licenses/by/4.0/
